# Alteration of the Ki-67 Proliferative Index following Surgical Resection with or without Radiation Therapy of Intracranial Meningiomas

**DOI:** 10.7759/cureus.1873

**Published:** 2017-11-23

**Authors:** Amir H Faraji, Daniel A Tonetti, John C Flickinger, Johnathan A Engh

**Affiliations:** 1 Department of Neurological Surgery, University of Pittsburgh Medical Center; 2 Department of Radiation Oncology, University of Pittsburgh Medical Center

**Keywords:** ki-67 labeling index, brain tumor, meningioma

## Abstract

The Ki-67 proliferative index is a widely accepted assay for cycling cells within tumor specimens of multiple histological subtypes. While it is not a substitute for the World Health Organization (WHO) grading, the Ki-67 proliferative index is thought to correlate with the biological activity of selected tumors. In the case of intracranial meningiomas, many lesions may be resected multiple times, with radiation therapy juxtaposed between surgical procedures. A retrospective review of 3,900 consecutive patients undergoing intracranial surgical resection at the University of Pittsburgh Medical Center over a five year period was undertaken. Of these patients, 604 had multiple resections. Multiple Ki-67 index scores were available for 42 patients with WHO grade I and II meningiomas, who suffered a recurrence or progression after their initial resection. Evidence of radiation therapy in the interval between pathology reports was also recorded. Data was evaluated for significant differences (p<0.05). WHO grade II meningiomas were more likely to have a higher Ki-67 index score on second resection than WHO grade I tumors (p=0.051). Furthermore, radiation-treated meningiomas demonstrated similar first Ki-67 index scores and higher second Ki-67 index scores (p=0.057 and p=0.022). Male patients tended to have less change in proliferation rates than female patients between the first and second resections (p=0.083), with a greater proportion of female patient tumors demonstrating accelerating proliferation rates. Treatment with radiation was associated with diminishing changes in meningioma proliferation rates compared to non-treated patients for tumors showing both accelerating rates (p=0.067) and decelerating rates (p=0.081). Ki-67 proliferation indices of recurrent or progressive meningiomas indicate that there are potentially distinct types of growth patterns of meningiomas, consisting of accelerating and decelerating proliferation rates. Meningioma growth is related to WHO grade, patient gender, and treatment with radiation. Radiation treatment appears to stabilize or “inactivate” tumor proliferation and thus normalize changes in meningioma growth rates.

## Introduction

Meningiomas are tumors arising from the coverings of the brain, which are typically benign but may result in significant morbidity depending on the location and size [1-2]. They are classified clinically according to the World Health Organization (WHO) grading scale and classification of brain tumors, which include grades I, II (atypical), and III (anaplastic) for meningiomas [2-4]. Several studies have examined the clinical and radiographic growth rates of these tumors to better understand the natural history of these lesions and their responses to treatment [5-13].

Just as the apparent growth rate of a tumor by radiological imaging is important, so too are changes in the molecular profile and proliferation rate of a tumor in response to stimuli. Ki-67 is an IgG_1_ class monoclonal antibody discovered by Gerdes, et al. in 1983 [14]. The antibody recognizes proliferating cells via a nuclear antigen that is absent in quiescent cells via immunostaining on fresh or frozen tissue. This antigen is expressed during all cell cycle phases, except for G_0_ and the early portion of G_1_. The discovery of the MIB-1 antibody, which also recognizes the Ki-67 antigen, allows immunostaining in formalin-fixed and paraffin-embedded tissue sections. A growing body of evidence suggests that the Ki-67 proliferative index may be suggestive of patient outcomes in patients with tumors, especially in lymphohematopoietic, locally invasive, and metastatic cancers [15-17]. While the function of the Ki-67 protein remains unclear in central nervous system tumors, its presence is used clinically to assess the proliferative index of tumors, for prognostic and diagnostic purposes [6, 18-25].

Among multiple brain tumor histopathological subtypes, the WHO grading represents a significant predictor of clinical outcome. However, clinical behavior is significantly varied even within these subtypes, and so additional markers may play a role, such as the Ki-67 proliferation index. Although there is some evidence that Ki-67 indices may be an independent predictor of clinical tumor behavior, it is unknown if tumor proliferation rates are altered by anti-tumor therapy, such as surgical resection and/or irradiation. In the present study we attempted to address a key question: does subtotal surgical resection of meningiomas, and/or treatment with radiation, correlate with changes in the Ki-67 proliferation rate? As an initial hypothesis, we expect WHO grade I meningiomas to demonstrate lower Ki-67 index scores on first and second surgical resections than WHO grade II meningiomas. Furthermore, we expect proliferation to be diminished in meningiomas treated with radiation. We will further analyze the proportion of tumors demonstrating accelerating and decelerating changes in proliferation rates in responses to surgical resection and treatment with radiation to aid in clinical decision-making and quantifying treatment responses in patients undergoing repeat surgical resection of their tumor(s).

## Materials and methods

Patient selection

We retrospectively identified 3,900 consecutive anonymized patients who underwent craniotomies at the University of Pittsburgh Medical Center over a five-year period. Of these patients, 110 had multiple tumor resections with at least two Ki-67 indices reported. Forty-two patients carried the general diagnosis of meningioma. Of these patients, 14 patients were male and 28 patients were female. Thirty-five of these meningiomas were WHO grade I and seven were WHO grade II. Anaplastic WHO grade III meningiomas were not included in our study. Pathology reports were obtained for these 42 patients, and the first and second Ki-67 indices, time interval between resections, WHO grade, and adjuvant treatment with radiation were recorded for each patient. All pathology reports were compiled from the faculty in the Neuropathology Division at the University of Pittsburgh Medical Center.

Data recording protocol

For all eligible tumors, Ki-67 proliferative indices were recorded. For cases in which a range was reported, the highest value was recorded in an effort to reduce bias towards slower-growing tumors. When the Ki-67 index was provided as a less than or greater than value, the percentage value provided was used. When the Ki-67 value was reported as occasional or few cells, 1% was recorded as the Ki-67 index value. The time interval between the first and second surgical resections was provided in months. Patient charts were also reviewed for the administration of radiation during the time interval between resections. Exact dosages and treatment volumes were recorded for all instances in which ionizing radiation was used, when available.

Statistical methods

SPSS 16.0 (SPSS Inc., Chicago, Illinois, USA) software was used for the statistical analysis. Multivariate regression with stepwise-forward linear and logistic regression was performed to identify significant variables that impacted Ki-67 index changes. Each of the histopathological subgroups was evaluated against relevant variables using one- or two-tailed Student’s t-tests, ANOVA with Tukey’s Honestly Significant Difference (HSD) post-hoc test, or Mann-Whitney U tests when appropriate due to small sample size or non-parametric data sets. Of note, using parametric statistical methods may overstate Type 1 error in instances of small sample sizes or data not normally-distributed. The results were analyzed for statistical significance (p<0.05). All data is presented as mean ± standard error of the mean (SEM).

## Results

The meningioma study group was composed of 42 patients with the histopathological diagnoses of WHO grade I (35 patients, 83.3%) and WHO grade II (seven patients, 16.7%) meningiomas. In total, 14 patients were male and 28 patients were female. The gender ratio breakdown for WHO grade I and WHO grade II subgroups were 12:23 and 2:5 male to female, respectively. The patient demographics and results on Ki-67 values, growth rates, and radiation treatment are provided as Table 1. No significant difference exists between the first and second Ki-67 index scores across all meningiomas (p=0.710).

**Table 1:** Patient Age, Average First and Second Ki-67 Index Scores, Time Interval Between Surgical Resections, and Changes in Proliferation Rates of Meningiomas by WHO Grade and Radiation Treatment

	Number of Patients		Patient Age (years)	First Ki-67 Index (%)	Second Ki-67 Index (%)		Time Interval (months)	Change in Proliferation Rate (Δ% per month)	p 1^st^:2^nd^ Ki-67
	All Patients	Radiation Treated							
All Patients									
All Patients	42	14	51.0 ± 2.5	9.2 ± 1.2	9.6 ± 1.3		16.1 ± 3.5	1.4 ± 1.4	0.710
World Health Organization (WHO) Grade									
WHO Grade I	35	10	52.0 ± 2.6	8.4 ± 1.2	8.1 ± 1.2	17.1 ± 3.7		0.5 ± 1.0	0.815
WHO Grade II	7	4	46.0 ± 7.0	13.3 ± 3.5	16.9 ± 4.5	11.0 ± 2.4		5.6 ± 7.2	0.219
p WHO I:II			0.235	0.113	0.051	0.208		0.256	
Radiation Treatment									
Yes Radiation	14		55.1 ± 3.8	11.9 ± 2.1	14.0 ± 2.8	20.0 ± 4.8		0.2 ± 0.3	0.356
No Radiation	28		49.2 ± 3.2	7.8 ± 1.3	7.4 ± 1.3	14.1 ± 4.7		2.0 ± 2.1	0.668
p Yes:No			0.124	0.057	0.022	0.193		0.198	

The effect of WHO grade on the Ki-67 scores of the 42 patients was analyzed; while there was no significant difference between the initial Ki-67 index scores of the WHO grade I and WHO grade II groups (p=0.113), a noteworthy trend for significance existed between the second Ki-67 index scores of the two groups, with WHO grade II grade meningiomas having a higher proliferative index (8.1 ± 1.2% versus 16.9 ± 4.5%, p=0.051). This result may be expected due to the typically aggressive nature of higher grade tumors. Although not statistically significant, WHO grade II meningiomas underwent re-resection approximately six months sooner than the WHO grade I meningioma group (p=0.208).

Furthermore, the effect of radiation treatment on the Ki-67 index scores of the 42 patients was analyzed. Again, while there was no significant difference between the initial Ki-67 index scores in the radiation-treated and non-treated groups (p=0.057), a significant difference was detected between the second Ki-67 index scores in the radiation-treated and non-treated groups (14.0 ± 2.8% versus 7.4 ± 1.3%, respectively, p=0.022) with the radiation-treated group having a higher second Ki-67 proliferation rate. The age of the patient and time interval between surgical resections did not vary statistically between the radiation-treated and non-treated groups (p=0.124 and p=0.193, respectively).

It may be postulated that there is divergence in the growth patterns of meningiomas prior to a second surgical resection that lead to a higher second Ki-67 proliferative index score, and that WHO grade and treatment with radiation may be correlated with this divergence. To better understand this finding, the change in proliferation rate of a tumor can be determined from the absolute difference between Ki-67 index scores and the time interval between resections. This change in proliferation rate may be correlated with accelerating or decelerating tumor growth in response to a treatment, for instance. The change in proliferation rate of all meningiomas was found to be 1.4 ± 1.4% per month, while WHO grade I and II were 0.5 ± 1.0% per month and 5.6 ± 7.2% per month, respectively. Despite this evidence that WHO II tumors may be proliferating at faster rates, there was no statistical difference found between the change in proliferation rates of the WHO grade I and II meningiomas; presumably, this is due to the large variances of the two groups and limited sample number (p=0.256). There was a trend in difference between the changes in proliferation rates in the meningiomas of male and female patients (-0.7 ± 0.8% per month and 2.4 ± 2.1% per month, respectively, p=0.085). This data is noteworthy, however, as males tended to not only demonstrate lower tumor proliferation rates than females, but that these changes in rates actually represented decelerated proliferation. Moreover, radiation-treated tumors demonstrated slowed growth as compared to non-treated tumors, though there was no statistical difference between the changes in proliferation rates of the radiation-treated and non-treated meningiomas as well (p=0.198). Again, this is presumably due to the limited sample size of this study and warrants further investigation.

As an independent approach to analyze the growth rates of meningiomas, two subgroups were devised from all tumors: tumors that demonstrated accelerating proliferation rates and decelerating proliferation rates. The data from this analysis is provided as Table 2. There was a significant difference between the two subgroups demonstrating accelerating and decelerating proliferation rates (p=0.026). Each group was divided with respect to patient age, gender, WHO grade, and radiation treatment to discover potential variables that correlated with accelerations or decelerations in tumor proliferation rates. No statistically significant correlative variables were identified; however, nearly significant trends for differences in the change in proliferation rate with patient gender and radiation treatment existed. There was no difference between the patient ages of the accelerating and decelerating proliferation rate subgroups (52.4 ± 3.8 years and 49.5 ± 4.7 years, respectively, p=0.324). Likely because of the limited sample size of the WHO grade II meningiomas, in particular, no correlative trend existed for WHO grade and changes in tumor growth rates, despite our reported finding that WHO grade II tumors may have a higher second Ki-67 index score on second resection.

**Table 2:** Comparing Meningiomas with Accelerating and Decelerating Proliferation Rates by Patient Gender, WHO Grade, and Radiation Treatment

	Accelerating Proliferation Rate		Decelerating Proliferation Rate	
	Percentage of Patients (%)	Acceleration Rate (Δ% per month)	Percentage of Patients (%)	Deceleration Rate (Δ% per month)
Patient Gender				
Male	21.4	1.2 ± 0.4	50.0	-1.8 ± 1.4
Female	46.4	7.1 ± 4.0	28.6	-3.1 ± 1.6
p – Accelerating Rate	0.083			
p – Decelerating Rate	0.284			
World Health Organization (WHO) Grade				
WHO I	37.1	3.6 ± 2.1	37.1	-2.1 ± 1.0
WHO II	42.9	16.4 ± 15.8	28.6	-5.0 ± 5.0
p – Accelerating Rate	0.251			
p – Decelerating Rate	0.331			
Radiation Treatment				
Yes Radiation	57.1	0.7 ± 0.3	28.6	-0.9 ± 0.6
No Radiation	28.6	11.2 ± 6.2	39.3	-3.1 ± 1.4
p –Accelerating Rate	0.067			
p – Decelerating Rate	0.081			

For meningiomas that demonstrated accelerating proliferation rates, male patients tended to demonstrate lower rates than female patients (p=0.083). There was no such trend for a difference in decelerating proliferation rates with patient gender (p=0.284). Of the 14 male patients with meningiomas, 21.4% were in the increasing growth rate subgroup and 50.0% were in the decreasing growth rate subgroup. In contrast, of the 28 female patients with meningiomas, 46.4% were in the accelerating proliferation rate subgroup and 28.6% were in the decelerating proliferation rate subgroup. Of note, a greater proportion of male patients underwent radiation treatment for their meningiomas than female patients. Nonetheless, these findings may correlate with the natural history and epidemiology of meningiomas, including the relationship between tumor growth and hormonal expression [26-28].

Furthermore, for meningiomas that demonstrated accelerating proliferation rates, patients treated with radiation demonstrated lower rates of growth acceleration than non-treated patients (p=0.067). In contrast, for meningiomas that demonstrated decelerating proliferation rates, patients treated with radiation demonstrated a decreased magnitude of proliferation rate change than non-treated patients (p=0.081). Therefore, it seems that radiation treatment of meningiomas serves to stabilize or “inactivate” tumor proliferation from accelerated or decelerated growth and thus to normalize the growth rate. This result supports previous reports of slowed radiographic growth rates in irradiated meningiomas [11-13, 29]. Of the 14 radiation-treated patients with meningiomas, 57.1% were in the accelerating proliferation rate subgroup and 28.6% were in the decelerating rate subgroup. In contrast, of the 28 non-treated patients with meningiomas, 28.6% were in the accelerating proliferation rate subgroup and 39.3% were in the decelerating rate subgroup. However, this result may be a product of selection bias, as meningiomas that progressed radiographically (and by extension, had accelerating tumor proliferation rates) were treated with radiation, and therefore a higher proportion of radiation-treated tumors exist in the accelerating proliferation rate subgroup.

## Discussion

While several seemingly contradictory results have been presented thus far: (1) meningiomas treated with radiation have significantly higher Ki-67 index scores on a second resection, and (2) it seems that radiation-treated tumors demonstrated slower growth than non-treated tumors. How may these results be reconciled? The divergence in tumor growth patterns may be related to the gender of the patient, WHO grade of the tumor, and/or radiation treatment. However, a plausible explanation is that meningiomas that progressed radiographically (and by extension, likely had higher Ki-67 proliferative rates) were treated with radiation, and therefore an increase in Ki-67 index scores may be related to selection bias. The trend towards slower growth of radiation-treated meningiomas may result from the radiation treatment itself. As an alternative hypothesis, tumors growing near their maximum biological potential have a higher probability to decrease their growth rate following an intervention. As noted in the growth rate analysis in the Results section, men had slower growing meningiomas than women; however, a greater proportion of men underwent radiation therapy than women. It should be noteworthy that the Ki-67 proliferative indices of meningiomas should not be viewed as static values. In contrast, the proliferation of a tumor likely varies depending on environmental or biological conditions. An analysis, as presented herein, which accounts for changes in the Ki-67 proliferation rate over time and in response to factors such as surgical resection and radiation therapy, may serve to better understand tumor progression in a longitudinal fashion. Work remains ongoing relating to initial tumor volume, residual post-resection volume, and treatment with radiation to provide further insight into Ki-67 proliferation rate changes.

## Conclusions

Meningiomas are typically benign growths of the linings of the brain, which usually grow slowly and cause morbidity due to mass effect on the surrounding brain parenchyma. In the case of these tumors, gross-total surgical resection may be curative with a substantially decreased chance of tumor recurrence. Our study is obviously biased towards tumors requiring a second resection, in order to observe changes in the Ki-67 proliferative index. In all, data relating to the Ki-67 proliferation indices of recurrent or progressive meningiomas indicate that there are three distinct types of growth patterns of meningiomas, consisting of accelerating, unchanging, and decelerating proliferation rates. Male patients tended to have blunted changes in proliferation rates than female patients, with a greater proportion of female patient tumors demonstrating accelerating proliferation rates. WHO grade II meningiomas were more likely to have a higher Ki-67 index score on second resection than WHO grade I tumors. Furthermore, radiation-treated meningiomas demonstrated higher Ki-67 index scores and likely correlated with clinical decision-making regarding the treatment of more aggressive tumors. Radiation treatment appears to stabilize or “inactivate” tumor proliferation and thus normalize changes in meningioma growth rates. As radiographically progressive or aggressive tumors, often in surgically inaccessible regions, are treated with radiation, this would be a desirable effect. Further studies on Ki-67 proliferation indices and changes in tumor proliferation rates are ongoing.

## References

[1] Chamoun R, Krisht KM, Couldwell WT (2011). Incidental meningiomas. Neurosurg Focus.

[2] Alahmadi H, Croul SE (2011). Pathology and genetics of meningiomas. Semin Diagn Pathol.

[3] Kleihues P, Burger PC, Scheithauer BW (1993). The new WHO classification of brain tumours. Brain Pathol.

[4] Louis DN, Ohgaki H, Wiestler OD (2007). The 2007 WHO classification of tumours of the central nervous system. Acta Neuropathol.

[5] Nakasu S, Fukami T, Nakajima M, Watanabe K, Ichikawa M, Matsuda M (2005). Growth pattern changes of meningiomas: long-term analysis. Neurosurgery.

[6] Hashimoto N, Rabo CS, Okita Y (2012). Slower growth of skull base meningiomas compared with non-skull base meningiomas based on volumetric and biological studies. J Neurosurg.

[7] Chang V, Narang J, Schultz L, Issawi A, Jain R, Rock J, Rosenblum M (2012). Computer-aided volumetric analysis as a sensitive tool for the management of incidental meningiomas. Acta Neurochir (Wien).

[8] Oya S, Kim SH, Sade B, Lee JH (2011). The natural history of intracranial meningiomas. J Neurosurg.

[9] Olivero WC, Lister JR, Elwood PW (1995). The natural history and growth rate of asymptomatic meningiomas: a review of 60 patients. J Neurosurg.

[10] Nakamura M, Roser F, Michel J, Jacobs C, Samii M (2003). The natural history of incidental meningiomas. Neurosurgery.

[11] Kondziolka D, Mathieu D, Lunsford LD, Martin JJ, Madhok R, Niranjan A, Flickinger JC (2008). Radiosurgery as definitive management of intracranial meningiomas. Neurosurgery.

[12] Kondziolka D, Nathoo N, Flickinger JC, Niranjan A, Maitz AH, Lunsford LD (2003). Long-term results after radiosurgery for benign intracranial tumors. Neurosurgery.

[13] Kondziolka D, Lunsford LD, Coffey RJ, Flickinger JC (1991). Stereotactic radiosurgery of meningiomas. J Neurosurg.

[14] Gerdes J, Schwab U, Lemke H, Stein H (1983). Production of a mouse monoclonal antibody reactive with a human nuclear antigen associated with cell proliferation. Int J Cancer.

[15] Faratian D, Munro A, Twelves C, Bartlett JM (2009). Membranous and cytoplasmic staining of Ki67 is associated with HER2 and ER status in invasive breast carcinoma. Histopathology.

[16] Hsi ED, Jung SH, Lai R (2008). Ki67 and PIM1 expression predict outcome in mantle cell lymphoma treated with high dose therapy, stem cell transplantation and rituximab: a Cancer and Leukemia Group B 59909 correlative science study. Leuk Lymphoma.

[17] Veltri RW, Isharwal S, Miller MC, Epstein JI, Mangold LA, Humphreys E, Partin AW (2008). Long-term assessment of prostate cancer progression free survival: evaluation of pathological parameters, nuclear shape and molecular biomarkers of pathogenesis. Prostate.

[18] Boker DK, Stark HJ (1988). The proliferation rate of intracranial tumors as defined by the monoclonal antibody KI 67. Application of the method to paraffin embedded specimens. Neurosurg Rev.

[19] Patsouris E, Stocker U, Kallmeyer V, Keiditsch E, Mehraein P, Stavrou D (1988). Relationship between Ki-67 positive cells, growth rate and histological type of human intracranial tumors. Anticancer Res.

[20] Ohta M, Iwaki T, Kitamoto T, Takeshita I, Tateishi J, Fukui M (1994). MIB1 staining index and scoring of histologic features in meningioma. Indicators for the prediction of biologic potential and postoperative management. Cancer.

[21] Ide M, Jimbo M, Yamamoto M, Umebara Y, Hagiwara S, Kubo O (1995). Growth rate of intracranial meningioma: tumor doubling time and proliferating cell nuclear antigen staining index. Neurol Med Chir (Tokyo).

[22] Sandberg DI, Edgar MA, Resch L, Rutka JT, Becker LE, Souweidane MM (2001). MIB-1 staining index of pediatric meningiomas. Neurosurgery.

[23] Aguiar PH, Tsanaclis AM, Tella OI, Jr. Jr., Plese JP (2003). Proliferation rate of intracranial meningiomas as defined by the monoclonal antibody MIB- 1: correlation with peritumoural oedema and other clinicoradiological and histological characteristics. Neurosurg Rev.

[24] Abry E, Thomassen IO, Salvesen OO, Torp SH (2010). The significance of Ki-67/MIB-1 labeling index in human meningiomas: a literature study. Pathol Res Pract.

[25] Heros RC (2012). Simpson Grade and MIB-1. J Neurosurg.

[26] Pines A (2011). Hormone therapy and brain tumors. Climacteric.

[27] Black PM (1997). Hormones, radiosurgery and virtual reality: new aspects of meningioma management. Can J Neurol Sci.

[28] Bondy M, Ligon BL (1996). Epidemiology and etiology of intracranial meningiomas: a review. J Neurooncol.

[29] Santacroce A, Walier M, Regis J (2012). Long-term tumor control of benign intracranial meningiomas after radiosurgery in a series of 4565 patients. Neurosurgery.

